# Paradoxical inflammatory switches during biologic therapy: a mechanistic framework, clinical algorithm, and pediatric illustrations

**DOI:** 10.3389/fped.2026.1797150

**Published:** 2026-05-20

**Authors:** Małgorzata Kowacka, Michał Dec, Hubert Arasiewicz

**Affiliations:** Clinical Department of Pediatric Dermatology and Vascular Anomalies, Faculty of Medical Sciences in Katowice, Medical University of Silesia, John Paul II Children’s and Family Health Center, Sosnowiec, Poland

**Keywords:** biologic therapy, immune rebalancing, JAK/STAT, Janus kinase inhibitors, paradoxical adverse events, paradoxical inflammation, pediatric dermatology, phenotypic switch

## Abstract

Paradoxical inflammatory reactions, also referred to as flip–flop phenomena, are increasingly recognized complications of biologic therapies targeting specific immune pathways. These reactions are characterized by the emergence of a new inflammatory phenotype with opposing immunologic polarization or by unexpected exacerbation of the underlying disease despite prior therapeutic response. Although numerous case reports have been published, a unified mechanistic framework and a practical clinical approach remain lacking, particularly in pediatric populations. In this narrative review, we synthesize current evidence on the immunopathogenesis of flip–flop reactions, focusing on dynamic interactions between the Th1/Th17, Th2, and interferon–JAK/STAT axes. We propose that paradoxical inflammation can represent a mechanistically consistent consequence of selective immune pressure rather than a coincidental adverse event. Five illustrative pediatric cases are presented as proof of concept, demonstrating distinct pathways leading to eczematous, psoriasiform, and interferon-driven phenotypes during biologic therapy. Based on mechanistic insights and clinical experience, we introduce a practical diagnostic and therapeutic algorithm designed to support early recognition and severity-adapted management of flip–flop reactions. Recognition of immune rebalancing as a therapeutic goal may facilitate rational treatment selection and improve outcomes in both pediatric and adult patients receiving biologic agents.

## Introduction

Paradoxical inflammatory reactions observed during biologic therapy represent a growing clinical challenge across immune-mediated diseases (1–3). These reactions—also described as paradoxical effects, overlap syndromes, or flip–flop phenomena—may involve the emergence of inflammatory conditions that are immunologically discordant with the disease being treated, or an unexpected worsening of the primary disease despite prior therapeutic response. In dermatology and rheumatology, this most often manifests as eczematous or psoriasiform eruptions arising during otherwise effective biologic therapy (2, 3). The increasing availability and earlier use of targeted biologic agents have expanded therapeutic options but simultaneously introduced new patterns of immune dysregulation (1, 3). While individual paradoxical reactions have been well documented, the prevailing literature remains fragmented and largely descriptive, offering limited mechanistic integration and limited guidance for clinicians faced with rapidly progressive or severe presentations (1, 2). Importantly, current conceptualizations often frame flip–flop reactions either as rare idiosyncratic adverse events or as coincidental coexistence of immune-mediated diseases (3, 4). Such interpretations do not fully account for recurring immunologic patterns observed across different drug classes and disease entities (1, 3). In this context, we propose that flip–flop phenomena reflect dynamic, system-level shifts in immune homeostasis induced by selective pathway inhibition. To address this, we conducted a targeted narrative review of the literature on paradoxical inflammatory reactions associated with biologic therapies, focusing on mechanistic studies, clinical reports, and pediatric observations relevant to immune-mediated inflammatory diseases. By integrating these data, we aim to provide a unified conceptual framework supported by illustrative pediatric cases, alongside a practical, severity-adapted diagnostic and therapeutic algorithm to support real-world clinical decision-making. Given the conceptual and hypothesis-generating nature of this work, the objective is to synthesize existing evidence within a systems-level framework rather than to perform a systematic analysis. To our knowledge, this is one of the first attempts to integrate paradoxical inflammatory reactions into a unified systems-level immunologic framework with direct clinical applicability.

## Immunologic homeostasis and targeted disruption

### The Th1/Th17 axis and targeted suppression

The Th1/Th17 axis plays a central role in the pathogenesis of psoriasis and juvenile idiopathic arthritis, driven primarily by TNF-α, IL-17, and IL-23 (5–11). These diseases represent some of the most common chronic inflammatory conditions encountered in dermatology and rheumatology (5, 6). Biologic therapies targeting these cytokines have demonstrated substantial clinical efficacy and have transformed disease management (6–9). However, profound suppression of dominant inflammatory pathways may disrupt immune equilibrium, creating conditions that favor activation of alternative immune axes (1, 9).

### The Th2 axis and eczematous inflammation

Th2-mediated inflammation, characterized by IL-4, IL-13, and IL-22 signaling, underlies atopic dermatitis and related eczematous disorders (12). Traditionally viewed as immunologically distinct from Th1/Th17-driven diseases, increasing evidence suggests partial overlap and plasticity between these inflammatory programs (13–16). Targeted blockade of Th1/Th17 pathways may unmask or amplify Th2-driven responses, particularly in predisposed individuals (3, 15, 16). These reactions can occur days to months after exposure to biologic agents and may persist for months after therapy withdrawal (3).

### The interferon–JAK/STAT axis as an amplifier

Type I interferons and downstream JAK/STAT signaling represent a critical interface between innate and adaptive immunity (17). Anti-TNF therapy, in particular, may lead to unchecked interferon production through loss of TNF-mediated inhibition of plasmacytoid dendritic cells (1, 17). This mechanism has been implicated in paradoxical inflammatory states, most frequently manifesting as psoriasiform eruptions, but also including hidradenitis suppurativa and other related inflammatory phenotypes (2, 18).

### Biologic therapy as selective immune pressure

**Figure 1:**
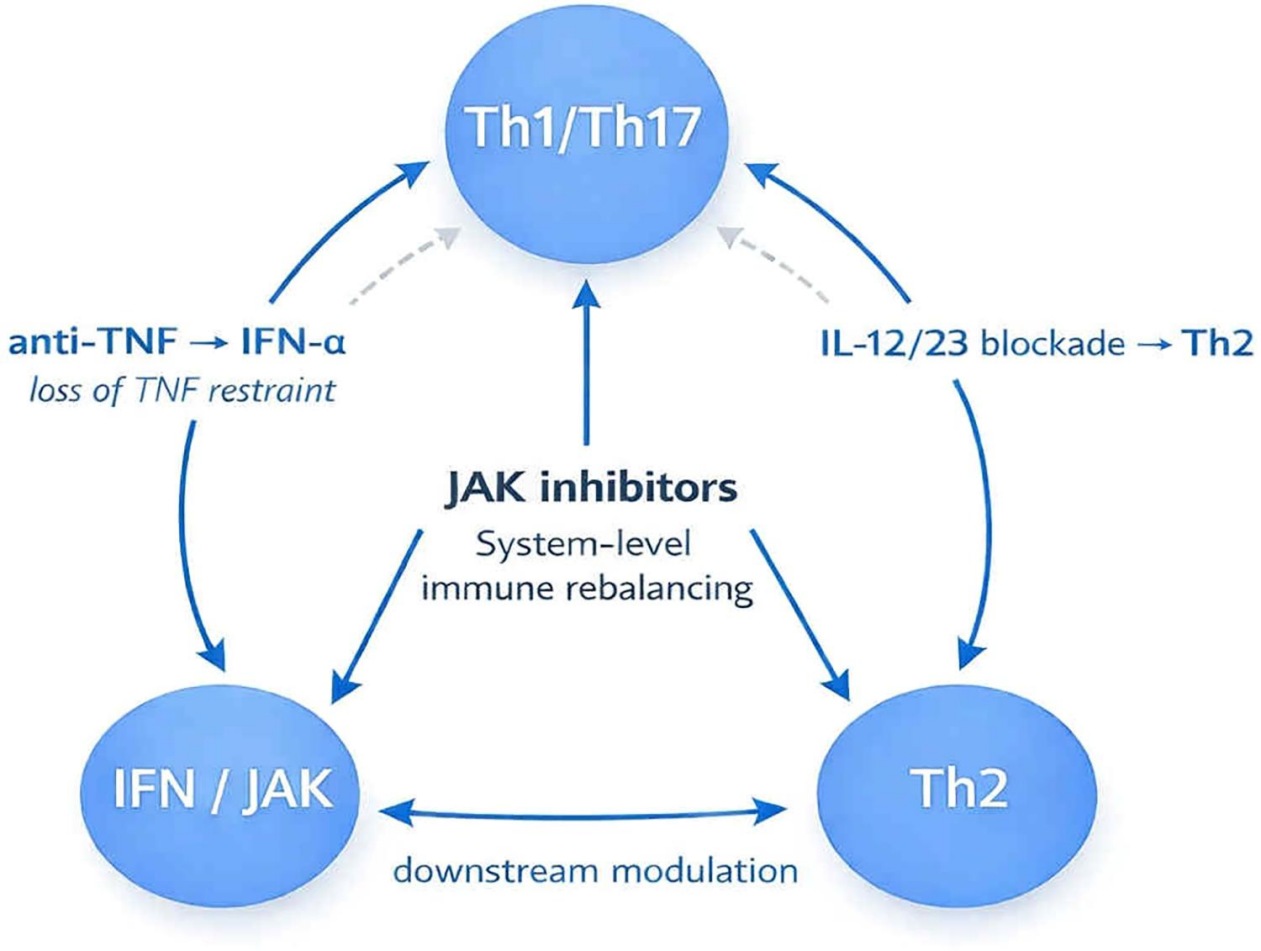
Immunologic flip–flop model and system-level immune rebalancing. Conceptual model illustrating paradoxical inflammatory (flip–flop) reactions during biologic therapy as a consequence of selective immune pressure. Targeted inhibition of dominant inflammatory pathways (e.g., TNF-α or the IL-12/23 axis) may destabilize immune homeostasis, leading to compensatory activation of alternative immune programs, including Th2-mediated inflammation or interferon-driven responses. Anti-TNF therapy may promote type I interferon signaling through loss of TNF-mediated regulation, whereas IL−12/23 blockade may favor Th2 polarization. The interferon–JAK/STAT axis functions as a central amplifier and integrator of these signals. Janus kinase inhibitors act downstream of multiple cytokine pathways, facilitating system-level immune rebalancing rather than pathway-specific suppression.

Rather than acting as global immunosuppressants, biologic agents exert directional immune pressure by selectively inhibiting specific cytokine pathways (1, 9). This selective modulation may destabilize immune homeostasis, leading to compensatory activation of alternative inflammatory circuits (3, 19–22). We propose that flip–flop reactions emerge from this imbalance and reflect immune reprogramming rather than coincidental pathology (1, 3, 21) (Figure 1).

### Flip–flop phenomena as a systems-level process

Flip–flop reactions should not be interpreted as simple coexistence of multiple inflammatory diseases (4, 15). Instead, they represent dynamic transitions between immune states driven by therapeutic intervention (1, 23, 24). The reproducibility of these patterns across drug classes and disease entities supports a systems-level mechanism rather than stochastic adverse events (1, 3). Clinical observations suggest that prior immune modulation, cumulative biologic exposure, and immunologic memory may lower the threshold for phenotypic switching (25). Once this threshold is crossed, inflammatory activity may rapidly shift toward an alternative axis, producing clinically distinct but mechanistically connected disease manifestations (15, 21). Combination therapy with biologic agents and conventional immunosuppressants has not been shown to prevent paradoxical inflammatory reactions (1). Accordingly, the aim of this framework is to support mechanistic reasoning and bedside decision-making rather than to derive epidemiologic estimates.

#### Pediatric-specific considerations

Pediatric patients represent a uniquely vulnerable population in the context of biologic-induced immune rebalancing. Immune regulatory networks in children are still developing, with reduced redundancy and greater plasticity compared to adults (25). This may predispose pediatric patients to more abrupt and severe phenotypic transitions when immune equilibrium is disrupted (25, 26). Furthermore, therapeutic options in pediatric populations remain limited, particularly with respect to newer biologics and small-molecule inhibitors (26, 27). As a result, flip–flop reactions in children may progress rapidly and require urgent, well-coordinated management strategies (27–29).

##### Illustrative pediatric cases (proof of concept)

All cases are anonymized and presented for illustrative purposes; formal ethics approval was not required for this narrative description in accordance with local regulations.

To illustrate these mechanisms, we present five pediatric cases demonstrating distinct flip–flop pathways. The first case involved a patient with psoriasis, a disease characterized by a dominant Th1/Th17 immune phenotype, in whom blockade of the IL-12/23 axis (ustekinumab) was associated with the rapid development of severe eczematous erythroderma, consistent with a shift toward Th2-predominant immune responses. Cutaneous lesions affected anatomical areas previously uninvolved by the underlying disease and developed within the first three months of biologic therapy. Marked clinical improvement was observed following discontinuation of the biologic agent and initiation of cyclosporine, supporting the reversibility of phenotypic switching upon modification of immune pressure. The second case concerned a patient with juvenile idiopathic arthritis and a positive atopic history, treated with combination therapy consisting of an anti-TNF agent (adalimumab) and methotrexate. After approximately one year of good treatment tolerance and clinical response, interferon-driven inflammation emerged, manifesting with clinical features consistent with hidradenitis suppurativa. The inflammatory process responded favorably to treatment with a Janus kinase inhibitor (tofacitinib), highlighting the central role of the IFN–JAK/STAT axis in the pathogenesis of this paradoxical reaction. The remaining three cases involved patients with Crohn's disease, two of whom had a documented history of atopy, treated with anti-TNF agents (adalimumab or infliximab). All developed cutaneous manifestations characteristic of an eczematous Th2-skewed phenotype, which were refractory to standard topical therapies, including calcineurin inhibitors and topical corticosteroids. The persistence and severity of skin involvement necessitated discontinuation of the biologic agents and initiation of Janus kinase inhibitors, further underscoring the role of secondary immune axis reprogramming in flip–flop reactions. These cases exemplify how selective pathway suppression may result in divergent but mechanistically predictable inflammatory outcomes (Table 1).

**Table 1:** Summary of illustrative pediatric cases.

Case	Underlying disease	Biologic therapy	Time to reaction	Phenotype	Management	Outcome
1	Psoriasis	Ustekinumab	∼3 months	Eczematous erythroderma	Discontinuation + cyclosporine	Improvement
2	JIA	Adalimumab + MTX	∼12 months	HS-like/IFN-driven	JAK inhibitor	Improvement
3	Crohn's	Adalimumab	Months	Eczematous	Discontinuation + JAK inhibitor	Improvement
4	Crohn's	Infliximab	Months	Eczematous	Discontinuation + JAK inhibitor	Improvement
5	Crohn's	Adalimumab	Months	Eczematous	Discontinuation + JAK inhibitor	Improvement

## Therapeutic implications: from suppression to rebalancing

Management of flip–flop reactions requires a shift in therapeutic thinking—from escalation of pathway-specific suppression toward restoration of immune balance (1, 3). Switching within the same biologic class may be insufficient to control paradoxical inflammation and can contribute to ongoing immune instability in selected cases (3, 18). Janus kinase inhibitors represent a mechanistically rational therapeutic alternative, as they simultaneously target multiple cytokine pathways (26, 30, 31). By modulating downstream components of inflammatory signaling rather than individual cytokines, these agents facilitate immune rebalancing and allow coordinated control of paradoxical inflammatory responses alongside the underlying disease process (23, 26, 30). Small-molecule inhibitors may be particularly advantageous in cases characterized by atypical or dynamically shifting inflammatory phenotypes, as they modulate JAK/STAT signaling independently of the dominant cytokine profile (23, 29, 32). Through their combined effects on the IL-23/IL-17 axis and Th2-driven immune responses, Janus kinase inhibitors enable broader regulation of immune networks (9, 17). Compared with conventional immunosuppressive therapies, they demonstrate a more rapid onset of action, supporting their use in time-sensitive clinical scenarios following the emergence of flip–flop reactions (28, 33, 34). Importantly, available clinical observations have not revealed significant differences in therapeutic response according to patient age or sex (29).

## Proposed diagnostic and therapeutic algorithm

**Figure 2:**
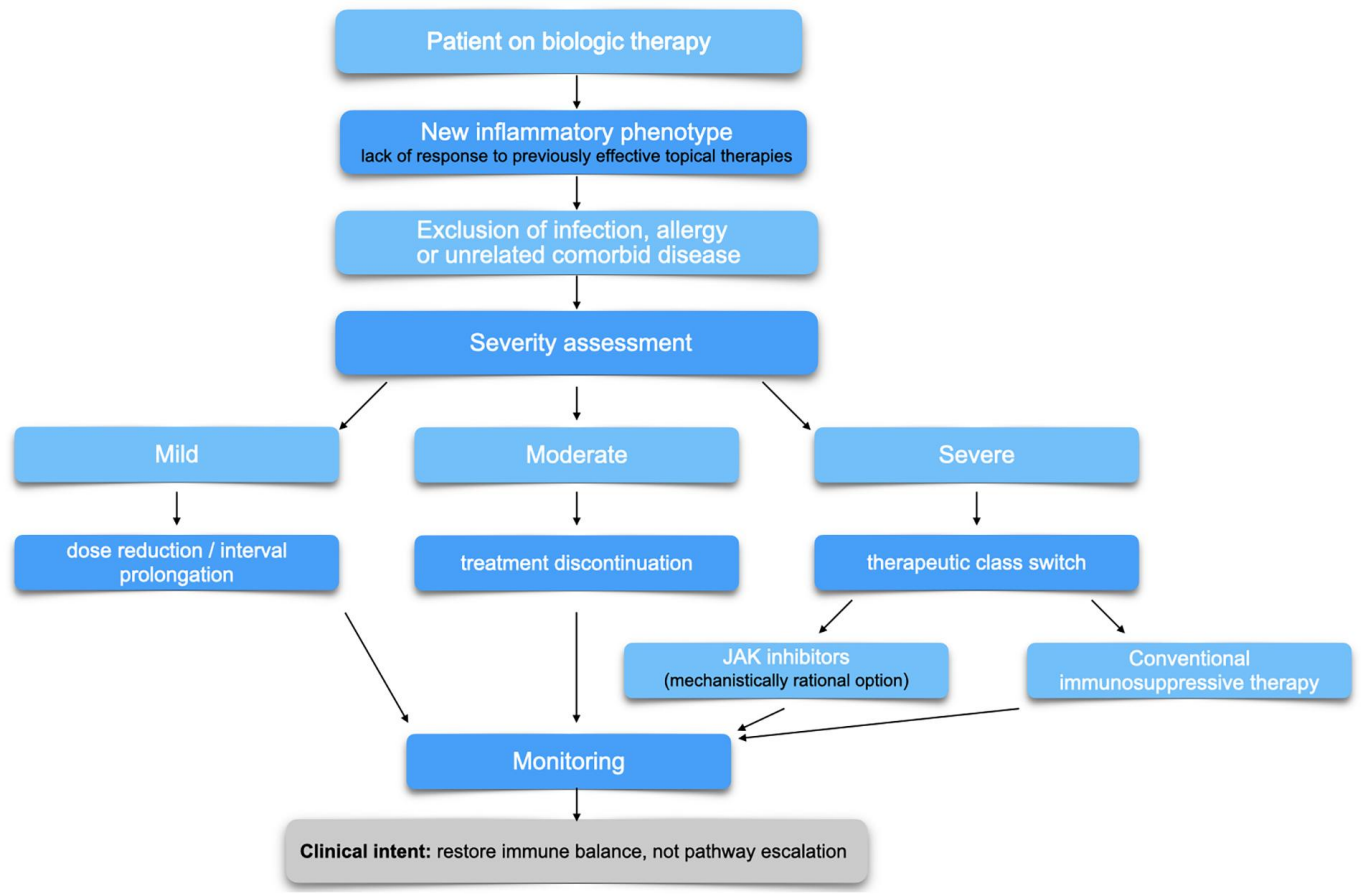
Severity-adapted diagnostic and therapeutic algorithm for paradoxical inflammatory reactions during biologic therapy. Proposed stepwise diagnostic and therapeutic algorithm for the recognition and management of paradoxical inflammatory (flip–flop) reactions in patients receiving biologic therapy. Following the emergence of a new inflammatory phenotype, alternative causes such as infection, allergy, or unrelated comorbid disease should be excluded. Management is guided by severity assessment, with mild cases allowing dose adjustment or interval prolongation, moderate cases prompting treatment discontinuation, and severe reactions necessitating therapeutic class switching. Janus kinase inhibitors represent a mechanistically rational option due to their capacity to modulate multiple inflammatory pathways, while conventional immunosuppressive therapies may be considered as alternatives. Ongoing monitoring and avoidance of frequent biologic or immunosuppressive switching are emphasized to support restoration of immune equilibrium.

Based on available evidence and clinical experience, we propose a stepwise diagnostic and therapeutic algorithm for flip–flop reactions (Figure 2). Early recognition, exclusion of alternative etiologies, severity assessment, and tailored modification of systemic therapy are central components. This approach is intended as a clinical reasoning tool rather than a formal guideline and emphasizes avoidance of repeated class switching while prioritizing strategies capable of restoring immunologic equilibrium. Prospective validation and real-world evaluation of this approach are warranted (33–35).

## Limitations and future directions

This framework is primarily based on observational data and illustrative cases, highlighting the need for prospective studies, biomarker development and translational immune profiling. Pediatric-specific registries and mechanistic studies are particularly warranted to refine risk stratification and therapeutic decision-making. The proposed framework remains hypothesis-driven and requires validation in prospective clinical and translational studies.

## Conclusions

Flip–flop phenomena may represent a predictable consequence of targeted immune modulation rather than rare or idiosyncratic adverse events. Recognition of immune rebalancing as a therapeutic objective may improve outcomes and guide rational treatment strategies in patients receiving biologic therapies.

### Scope statement

This manuscript fits within the scope of pediatric dermatology and pediatric immune-mediated inflammatory disease research by addressing paradoxical inflammatory (flip–flop) reactions emerging during biologic therapy. The review integrates mechanistic immunology with pediatric clinical observations to propose a systems-level framework centered on interactions between Th1/Th17, Th2, and interferon–JAK/STAT axes. It further provides a practical severity-adapted diagnostic and therapeutic algorithm to support real-world clinical decision-making in children experiencing phenotypic switching under targeted therapies.

## Data Availability

The original contributions presented in the study are included in the article/Supplementary Material, further inquiries can be directed to the corresponding author.
